# A Telerehabilitation Intervention for Youths With Arthrogryposis Multiplex Congenita: Protocol for a Pilot Study

**DOI:** 10.2196/18688

**Published:** 2020-06-26

**Authors:** Marianne Gagnon, Jessica Collins, Caroline Elfassy, Gabriela Marino Merlo, Jacquelyn Marsh, Bonita Sawatzky, Rita Yap, Reggie Hamdy, Louis-Nicolas Veilleux, Noémi Dahan-Oliel

**Affiliations:** 1 Department of Surgery McGill University Montreal, QC Canada; 2 Shriners Hospital for Children-Canada Montreal, QC Canada; 3 School of Physical and Occupational Therapy McGill University Montreal, QC Canada; 4 School of Physical Therapy Western University London, ON Canada; 5 Department of Orthopedics University of British Columbia Vancouver, BC Canada; 6 Division of Paediatric Orthopaedics Department of Paediatric Surgery Montreal Children Hospital Montreal, QC Canada

**Keywords:** telerehabilitation, arthrogryposis multiplex congenita, physical therapy, occupational therapy

## Abstract

**Background:**

Arthrogryposis multiplex congenita (AMC) is characterized by joint contractures present in at least two body areas. In addition to these contractures, individuals with AMC can have decreased muscle mass, leading to limitations in activities of daily living. Exercise has the potential to maintain or improve the range of motion and muscle strength. However, this type of intervention necessitates frequent follow ups that are currently difficult to provide for youths with AMC because they often live far from a specialized hospital. To overcome this distance challenge, telecommunication technologies can be used to deliver rehabilitation remotely, which is called telerehabilitation. The study protocol for one such type of rehabilitation will be presented in this paper.

**Objective:**

This pilot study aims to (1) evaluate the feasibility of using telerehabilitation to provide a home exercise program for youths with AMC, and (2) assess the effectiveness of a home exercise program.

**Methods:**

A total of 10 youths aged 8-21 years with AMC will be recruited. The intervention consists of a 12-week individualized home-based exercise program delivered remotely using telerehabilitation. At baseline, youths will complete the Physical Activity Questionnaire for Adolescents and the Pediatrics Outcomes Data Collection Instrument to assess pain, function, and level of physical activity. During the first telerehabilitation meeting, the rehabilitation therapists will measure range of motion using a virtual goniometer and assess the youth’s functional level. The therapists will then use the Goal Attainment Scale to set objectives and develop the individualized intervention. Follow ups will occur every 3 weeks to make sure exercises are performed safely and to progress the exercises when needed. At the end of the 12-week intervention, rehabilitation therapists will re-evaluate the youth using the same outcome measures as the initial evaluation. The youths will be asked to complete the same questionnaires, with the addition of questions about their satisfaction regarding the intervention. Nonparametric and descriptive statistics will be used to evaluate the feasibility and effectiveness.

**Results:**

Ethics approval was obtained in October 2018. Recruitment and data collection started in January 2019 and was completed in May 2020.

**Conclusions:**

This pilot study will help us learn how a large-scale project may work in practice to improve outcomes in physical activity, pain, and function, and goal attainment among youths with AMC, thus informing a future clinical trial.

**International Registered Report Identifier (IRRID):**

DERR1-10.2196/18688

## Introduction

Arthrogryposis multiplex congenita (AMC) or arthrogryposis is a term representing a heterogeneous group of over 400 disorders characterized by congenital contractures present in at least two different body joints [1]. AMC is rare and affects 1 in 3000 to 1 in 4300 live births [2]. Amyoplasia and distal arthrogryposis are the most common types and combined together, they represent about 50%-65% of all AMC diagnoses [3,4]. Contractures, which are defined as the limitation of passive movement of a specific joint, can affect the joints of the upper and/or lower limbs, as well as the spine and jaw [1]. Contractures can be caused by an increase of connective tissue around the joints, joint fibrosis replacement of muscle, muscle atrophy, or articular deformities [1]. AMC is typically nonprogressive, as no new contractures appear during an individual’s life. However, contractures can recur after intervention or worsen over time [5]. In addition to these contractures, individuals with AMC may present with decreased muscle mass and bone density, bone deformities, and pain, leading to activity limitations and participation restrictions in activities of daily living such as feeding, dressing, mobility, and sports [3,6-9]. Early interventions such as stretching, splinting, bracing, casting, rehabilitation, and surgical procedures have been shown to augment and maintain range of motion and strength, and therefore, to promote independence in daily activities [10].

Rehabilitation can improve physical function and maintain gains after surgery [5]. Structural changes of the joint surface in AMC further limit range of motion when the joint is not used [10]. Besides, a positive association has been reported between knee and hip muscle strength and motor function [11], suggesting that rehabilitation exercises can maintain or increase range of motion and preserve muscle strength for optimal motor function. Currently, most interventions in AMC, specifically rehabilitation, occur in early childhood and their frequency decreases during school-age and adolescent years, despite new challenges arising during these transition periods [5,12]. Rehabilitation for school-aged children focuses mostly on body functions and structure, which does not always correspond to the youth’s specific needs, such as participating in activities [12].

As AMC is rare, youths are mainly treated in subspecialized health care centers, which may be geographically distant from where they live. Therefore, clinicians face an important challenge in implementing regular exercise interventions. Novel intervention approaches and technologies are needed to increase access to subspecialized care for youths with AMC across geographical boundaries. Telerehabilitation, defined as “an innovative way to deliver rehabilitation services remotely using information and telecommunication technologies” [13], can be used to overcome this challenge. Some aspects can limit the usability of telerehabilitation, such as the lack of direct contact with participants, difficulties with manipulation of technology for individuals with physical limitations, or having a poor internet connection [14,15]. Nevertheless, telerehabilitation has been studied with different clinical populations (eg, total knee arthroplasty or vascular surgery patients) and was found to be as effective as face-to-face interventions [13], save travel time [16], and reduce cost for people living at a distance of 30 km or more from the health care center [17]. In the pediatric population, telerehabilitation has been used to provide various interventions (physical therapy, psychology, and speech language therapy) for different populations (patients with acquired brain injury, autism spectrum disorder, or cerebral palsy) [18]. Despite the potential benefits of telerehabilitation, there is a lack of research on its use for youths with physical impairments [18]. Therefore, the purpose of this study is to pilot the delivery of a home-based exercise program (HEP) for youths with AMC using telerehabilitation. Specifically, this pilot study aims to (1) evaluate the feasibility of using telerehabilitation to provide a physical assessment and to deliver an HEP for youths with AMC; and (2) explore the potential effectiveness of the HEP on goal attainment, physical activity, pain, and function. The provision of an HEP through telerehabilitation will provide an unprecedented opportunity for service equity to this vulnerable population, regardless of geographical location. The methodology of this study is reported in this paper.

## Methods

### Study Design

This is a pilot study to evaluate the feasibility and effectiveness of a telerehabilitation intervention for youths with AMC.

### Participants Eligibility

Youths aged between 8 and 21 years with a confirmed clinical diagnosis of multiple congenital contractures, or AMC, will be invited to participate. Inclusion criteria include the ability to communicate in English or French and residence in Canada. Youths living in another country will be excluded as rehabilitation therapists participating in this study hold only a Canadian professional license. To align with postintervention precautions, those having undergone a recent surgery (ie, 3 months for soft tissue and 6 months for bony surgery) will be excluded. Youths with cognitive deficits or unstable health will also be excluded, to ensure participants can take part in the HEP. To assess the level of cognition, medical records will be reviewed for the presence of central nervous involvement or intellectual impairment. In addition, the treating physician, therapists, and parents will be consulted to determine eligibility based on type of schooling and ability to follow instructions. No restriction will be made about the degree of severity of contractures or physical impairment.

### Recruitment

Youths with AMC followed at Shriners Hospital for Children - Canada (SHC-C) will be recruited during their clinic visit. Potential youths with the appropriate clinical diagnosis who do not have an upcoming visit to SHC-C will be contacted by postal mail and phone. For youths with AMC who are not followed at SHC-C, an advertisement describing the study will be posted on social media of a Canadian AMC support group (ie, Facebook). Those interested in participating will be asked to contact the clinical research coordinator for more information. Prior to participating in the study, informed consent will be sought from parents as well as from youths aged 14 years and older, as per provincial regulations. Youths between the ages of 8 and 13 years will be asked to provide assent. For these younger participants, the research team will encourage parents to be available during the telerehabilitation sessions to provide support during the sessions and throughout the HEP. As we expect to complete this study on 10 youths, 13 youths will be recruited to account for a 30% dropout rate [19].

### Intervention

A rehabilitation team including a physical therapist, an occupational therapist, and a physical rehabilitation therapist will collaborate to provide the intervention. A physical rehabilitation therapist is defined as a professional among the interdisciplinary team whose role is to obtain necessary prerequisite information from a leading physical therapist in order to develop treatment plans and provide appropriate interventions to reduce activity limitations and participation restrictions.

**Figure 1:**
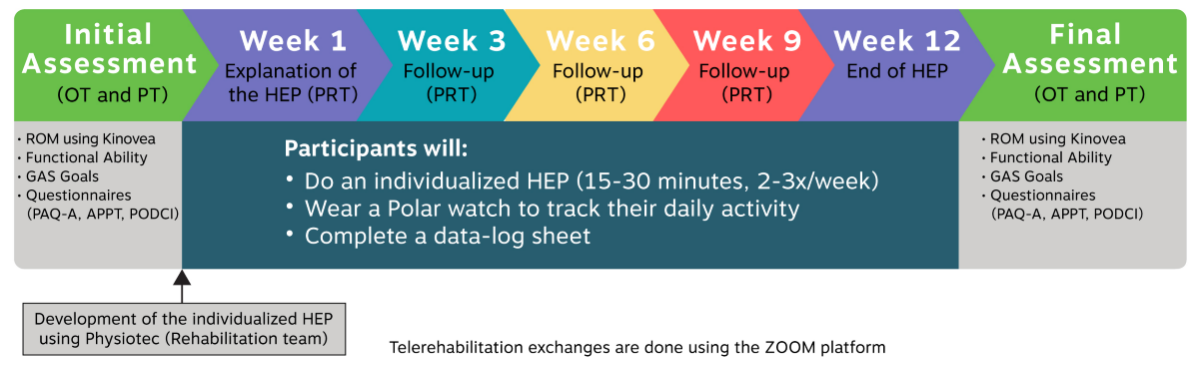
A summary of the 12-week telerehabilitation intervention. APPT: Adolescent and Pediatric Pain Tool; GAS: Goal Attainment Scale; HEP: home-based exercise program; OT: occupational therapist; PAQ-A: Physical Activity Questionnaire for Adolescents; PODCI: Pediatrics Outcomes Data Collection Instrument; PRT: physical rehabilitation therapist; PT: physical therapist.

The intervention will consist of a 12-week HEP. Prior to the assessment with the therapists, youths will complete an online questionnaire consisting of the Physical Activity Questionnaire for Adolescents (PAQ-A), the Pediatrics Outcomes Data Collection Instrument (PODCI), and demographic questions. The occupational therapist and physical therapist will conduct the initial assessment of each youth using ZOOM Pro (Zoom Video Communications, Inc), a videoconferencing platform allowing an encrypted connection. During this assessment, the therapists will perform an active joint range-of-motion (AROM) assessment of the upper and lower limbs, evaluate overall function, assess pain using the Adolescent and Pediatric Pain Tool (APPT), and establish individualized goals with each youth using the Goal Attainment Scale (GAS). The information gathered through this initial assessment will be used by the rehabilitation team to develop an individualized 12-week HEP. A week after the initial assessment, the physical rehabilitation therapist will exchange with the youth using ZOOM to explain and deliver the HEP as well as ensure comprehension and safe execution of the exercises. Youths will be asked to perform their HEP three times a week for approximately 15-30 minutes each time. They will be sent a physical activity monitor (POLAR watch A370) by postal mail and will be instructed to wear it on their wrist during HEP sessions, as it will be used to capture heart rate and the duration of the exercise sessions. Youths will have the possibility to wear the physical activity monitor at all times if they desire. If needed for the HEP, exercise materials such as resistant elastic bands or TheraPutty will also be sent to them by postal mail. A follow up with the physical rehabilitation therapist will be provided through ZOOM every 3 weeks (ie, at weeks 3, 6, and 9) to address any questions and to adjust the HEP as needed. At the end of the 12-week intervention, the occupational therapist and physical therapist will re-evaluate the youth using the same outcome measures as the initial evaluation. Youths will be asked to complete the same questionnaire, with the addition of questions about their satisfaction regarding the intervention. Figure 1 provides a summary of the intervention. The outcome measures used in this study are described in the following sections. In addition, parents will be asked to complete a cost questionnaire (direct and indirect costs incurred in relation to their child’s condition), which will be described elsewhere as it extends beyond the scope of this study.

### Measurement

#### Feasibility

##### Operationalization

The source of recruitment (eg, in clinic, postal mail, phone, or social media), recruitment and withdrawal rates, compliance to the HEP and to the telerehabilitation meetings, and missing data will be calculated to determine feasibility of the intervention for youths with AMC. The compliance to the HEP will be measured using the data from the activity monitor as well as from a data-log sheet on which youths will have to record when they perform their exercise program. Refer to Table 1 for the operationalization of the feasibility criteria.

**Table 1:** Operationalization of the criteria to evaluate feasibility.

Parameters	Definition	Criteria/examples
Source of recruitment	The method used to recruit each youth.	In clinic, postal mail, phone, social media
Recruitment rates	From a list of patients with AMC^a^ followed, the number of eligible youths will be determined. Those that are reachable will be accounted.	≥50% of eligible and reachable youths
Withdrawal rates (before the intervention)	Youths who will consent to participate but will withdraw before the start of the intervention will be accounted.	≤20% of the youths who consent
Withdrawal rates (during the course of the intervention)	Youths who will complete at least one telerehabilitation meeting and will decide to withdraw afterward will be counted. The time points when they decide to withdraw will be collected as well as the reason, if applicable.	≤30% of the youths who start the intervention
Completion rates	The number of youths who will complete all 6 telerehabilitation meetings out of those who have consented.	≥50% of the youths who consent
Compliance to the HEP^b^	The amount of time youths performed their HEP will be collected using a participant-completed log sheet and data from the physical activity monitor.	≥50% of compliance to the HEP
Compliance to the telerehabilitation meetings	The number of meetings cancelled the same day among the meetings that occurred, as well as lateness to the meetings. Lateness is defined as joining the meeting 15 minutes or more after the scheduled time.	≤15% of the meetings
Missing data	The number of questionnaires not completed and the number of unusable range-of-motion data.	≤10% for each outcome
Technical issues	Problems that will arise and disrupt or delay the meeting or possibly prevent the telerehabilitation meeting from taking place.	Echo voices, connection, image quality

^a^AMC: arthrogryposis multiplex congenita.

^b^HEP: home-based exercise program.

##### Cost

The total amount of time spent during the telerehabilitation intervention by the physical therapist, occupational therapist, and the physical rehabilitation therapist providing the assessment and the HEP as well as their time outside the direct intervention with the youth (planning the HEP and writing the report) will be recorded. The cost of the ZOOM plan and of the federal professional licenses for the occupational therapist and physical therapist will be reported.

##### Satisfaction

Satisfaction regarding the telerehabilitation intervention, the assessment with the therapists, and the HEP will be evaluated in the final online questionnaire with open-ended questions and using a 5-point Likert scale.

#### Effectiveness

##### GAS

The GAS will be administered by the occupational therapist with the youth during the initial online assessment to set individualized goals with the youths and to calculate the extent to which their goals are met at the end of the 12-week HEP [20].

##### AROM

AROM will be measured to provide an overview of the youths’ ability to build the HEP. Screenshots of each youth performing specific movements during the initial and final assessment will be taken and then AROM will be measured using a virtual goniometer (Kinovea). This method has been shown to be feasible on 10 healthy adult participants [21]. AROM of the following joints will be measured as degrees of movement: shoulder (abduction, flexion, and extension), elbow (flexion and extension), forearm (pronation and supination), wrist (flexion and extension), hip (flexion, extension, internal rotation, and external rotation), knee (flexion and extension), and ankle (dorsiflexion and plantar flexion). Some movements are expected to be difficult to measure with a virtual goniometer because of the wrong plane of movement, so we will report the following AROM as full, limited, or absent: finger and thumb (flexion, extension, abduction, and adduction), shoulder (internal and external rotation), and hip (abduction). When possible, AROM will be measured in positions that allow being with and without the effect of gravity to estimate muscle strength. Although passive range of motion is usually taken clinically, it will not be measured in this context of telerehabilitation.

##### PODCI

This questionnaire was developed by the American Academy of Orthopaedic Surgeons and the Pediatric Orthopaedic Society of North America to measure functions in the following dimensions among children aged 2-18 years with musculoskeletal conditions: upper extremity functioning, transfers and basic mobility, sports and physical function, and comfort/pain [9]. The four dimensions are computed together to give a global functioning score, and in addition, a separate scale evaluating happiness with physical condition is provided. The PODCI response scales use a 3- to 6-point format. The internal consistency is high and the test–retest reliability is excellent for all subscale scores [22]. The PODCI was shown to be sensitive to change in function over time in 74 children with amyoplasia [9].

##### PAQ-A

This questionnaire measures the general levels of physical activity in the last 7 days. The PAQ-A is a self-administered questionnaire developed for typically developing high-school-age youths. This questionnaire has shown a moderately high concurrent validity when compared with an activity monitor and a good internal consistency [23]. The PAQ-A provides a summary physical activity score derived from 8 items, each scored on a 5-point scale. The final mean of the 8 items is reported to classify the level of physical activity [24].

##### Adolescent Pediatric Pain Tool

This tool assesses current pain for children and adolescents between ages 8 and 17 years with different conditions such as cancer and orthopedic and traumatic injuries. The APPT shows good validity, test–retest reliability, and sensitivity. The questionnaire uses a visual analog scale, a list of 42 qualitative words describing pain, and 2 body diagrams on which the child has to circle the location of the pain [25]. A summary of the outcomes to assess the effectiveness is presented in Table 2.

**Table 2:** Summary of outcomes used to determine effectiveness.

Tools/outcome measures	What does it assess?
Adolescent Pediatric Pain Tool (APPT)	Pain
Goal Attainment Scale (GAS)	Progress of the individualized goals
Physical Activity Questionnaire for Adolescents	Level of physical activity
Pediatrics Outcomes Data Collection Instrument	Upper extremity function Transfers and basic mobility Sports and physical function Comfort and pain Global function Happiness
Range of motion	Shoulder: abduction, adduction, flexion, and extension Elbow: flexion and extension Forearm: pronation and supination Wrist: flexion and extension Hip: flexion, extension, internal rotation, and external rotation Knee: flexion and extension Ankle: dorsiflexion and plantar flexion

### Statistical Analyses

Given this is a pilot study with a small sample size and a heterogeneous population, nonparametric and descriptive statistics will be used. To assess feasibility, the compliance to the HEP and to the telerehabilitation meetings, recruitment and completion rates, missing data, and closed-ended questions on satisfaction will be analyzed using descriptive statistics and compared with the established feasibility criteria. Table 1 describes the feasibility criteria for operationalization that will be used in the study. Youths’ experience about use of technology and overall satisfaction with the program will be reported as collected in the open-ended questions. The cost of the telerehabilitation intervention (ie, therapists’ time, federal professional license, and teleconferencing cost), recruitment source, and technical issues experienced during the program will be described. For the effectiveness of the HEP, raw GAS scores will be converted to GAS T-scores for each youth and for the global HEP. Preintervention and postintervention data of the range of motion, APPT, PAQ-A, and PODCI will be compared using the Wilcoxon signed-rank test to measure change among the same youth.

## Results

Administrative site approval was obtained from the Department of Medical Research at Shriners Hospital for Children (CAN1806) in July 2018. Ethics approval was provided by the McGill University Faculty of Medicine Institutional Review Board (#A08-B38-18B) in October 2018. Recruitment and data collection started in January 2019 and was completed in May 2020.

## Discussion

### Overview

The proposed telerehabilitation pilot study is designed to provide an HEP to youths with AMC. Establishing the feasibility of this study has the potential to inform service delivery models using technology, thereby overcoming geographical barriers, to provide an individualized HEP to youths living with a rare condition.

To date, little is known about the potential effectiveness of exercises in adolescents with AMC [5]. Using this type of technology in a study that includes frequent follow ups as proposed in this protocol has the potential to reach more participants because it transcends geographical barriers, improving the sample size needed for research with heterogeneous populations such as AMC. Telerehabilitation allows inclusion of youths from across Canada, regardless of proximity to a specialized health care center. Although telerehabilitation has previously been used in studies on children with asthma and autism spectrum disorders [26,27], the only musculoskeletal disorder in which a telerehabilitation intervention was used is with children with cerebral palsy [18]. However, the results were variable among those studies which justify studies with other populations such as AMC [28,29]. In addition, little is known about the cost efficiency of this type of intervention [26,30].

### Limitations

This pilot study has some limitations. Because of the remoteness of the assessment, even if passive range of motion is normally measured in the clinic, it will not be assessed in this study. Despite passive range of motion being important to measure, it is expected that therapists will have sufficient information to develop the individualized HEP from the initial telerehabilitation assessment. Another consideration is that youths will be instructed to wear the activity monitor on their wrists, even in the presence of upper extremity contractures that may interfere with the movement capture, therefore with the accuracy of step count. As the purpose of the activity monitor in this study is to measure heart rate and duration of the exercise sessions, and not to record steps, all youths will wear the watch at the wrist. Importantly, the goal of using telerehabilitation is not to replace face-to-face clinic visits or in-person therapy, but rather to propose a complementary intervention therapists can offer youths and their families during adolescence, at which time rehabilitation has been shown to decrease [12]. The aim to provide an individualized HEP is to help youths maintain their physical gains and assist them in reaching new goals that may arise as they grow. This telerehabilitation pilot study will also inform the possible pitfalls, beneficial effects, and cost associated with this new method of care, thus informing its use in other populations with musculoskeletal conditions.

### Conclusion

Establishing the feasibility of using telerehabilitation for children with AMC will inform us for a future clinical trial. Information about the potential effectiveness of using telerehabilitation to deliver an HEP for children with AMC will be provided, thus leading to the development of a novel approach in this population. If proven successful, this service delivery model can be tailored to other pediatric musculoskeletal conditions, such as osteogenesis imperfecta and juvenile rheumatoid arthritis.

## Abbreviations

AMC: arthrogryposis multiplex congenita
APPT: Adolescent and Pediatric Pain Tool
AROM: Active range of motion
GAS: Goal Attainment Scale
HEP: Home-based exercise program
PAQ-A: Physical Activity Questionnaire for Adolescents
PODCI: Pediatrics Outcomes Data Collection Instrument
SHC-C: Shriners Hospital for Children - Canada
